# Impact of surgical volume and specialist availability on thyroidectomy outcomes in Brazil: an ecological study with a nationwide retrospective analysis of 230,345 cases (2008–2023)

**DOI:** 10.1590/1516-3180.2025.2938.11112025

**Published:** 2026-04-10

**Authors:** Ricardo Yugi Eri, Leandro Luongo Matos, Marcelo Passos Teivelis, Nelson Wolosker, Ana Kober Nogueira Leite

**Affiliations:** I Medical Resident, Hospital Albert Einstein, São Paulo (SP), Brazil.; II Full Professor, Faculdade Israelita de Ciências da Saúde Albert Einstein, Hospital Albert Einstein, São Paulo (SP), Brazil; Departamento de Cirurgia de Cabeça e Pescoço, Instituto do Câncer do Estado de São Paulo, Hospital das Clínicas, Faculdade de Medicina, Universidade de São Paulo (HC-FMUSP), São Paulo (SP), Brazil.; III Assistant Professor, Faculdade Israelita de Ciências da Saúde Albert Einstein, Hospital Albert Einstein, São Paulo (SP), Brazil.; IV Dean, Faculdade Israelita de Ciências da Saúde Albert Einstein, Hospital Albert Einstein, São Paulo (SP), Brazil.; V Assistant Professor, Faculdade Israelita de Ciências da Saúde Albert Eintein; Instituto e Pesquisa Albert Einstein, Hospital Albert Einstein, São Paulo (SP), Brazil; Departamento de Cirurgia de Cabeça e Pescoço, Instituto do Câncer do Estado de São Paulo, Hospital das Clínicas, Faculdade de Medicina, Universidade de São Paulo (HC-FMUSP), São Paulo (SP), Brazil.

**Keywords:** Thyroid, Thyroidectomy, Cancer of head and neck, Specialist, Surgeon, Head and neck, Thyroid disease, Public health, Epidemiology, Medical specialization

## Abstract

**INTRODUCTION:**

Thyroid disorders affect a significant proportion of the population and often necessitate surgical intervention, particularly in cases of malignancy. In this study, we analyzed thyroidectomy procedures performed within the public health system (SUS) of Brazil from 2008 to 2023, with a focus on factors influencing in-hospital mortality.

**METHODS:**

In the retrospective analysis, data on 230,345 thyroidectomies were extracted from SUS records and stratified by hospital volume and state-level distribution of head and neck specialists. Mortality rates were evaluated using non-parametric statistical analyses, including the Kruskal–Wallis and Spearman’s correlation tests.

**RESULTS:**

The overall in-hospital mortality rate was 0.15%. States with fewer than 0.5 head and neck specialists per 100,000 inhabitants exhibited significantly higher mortality rates (0.2 versus 0.15, p = 0.02). Hospitals performing fewer than 25 procedures annually (very low-volume) had a fivefold increase in mortality compared with high-volume hospitals (0.51 versus 0.10, p < 0.001). When hospitals performing fewer than 10 procedures per year were excluded, significant differences in mortality among volume groups were no longer observed.

**CONCLUSION:**

Mortality increased significantly in very low-volume hospitals performing fewer than 25 thyroidectomies per year; however, this difference was no longer observed after excluding hospitals that performed fewer than 10 interventions per year. These results support restricting thyroidectomies to hospitals performing at least 10 procedures annually and promoting centralization to improve outcomes. Structured regionalization policies are needed to ensure equitable access to specialized surgical care across Brazil.

## INTRODUCTION

 Thyroid disorders are prevalent in Brazil, affecting as much as 12% of the population.^1^ These conditions encompass a broad spectrum of thyroid disorders, including hypothyroidism, hyperthyroidism, goiter, nodules, thyroiditis, and malignancies. Although while many thyroid disorders can be effectively managed with medical treatment, thyroidectomy remains the cornerstone of surgical intervention for several benign and most malignant thyroid conditions.^2^

 In recent decades, the global incidence of thyroid cancer has significantly increased. This increase is attributed to the widespread use of advanced medical imaging techniques, allowing the detection of incidental thyroid cancers that otherwise would have remained unnoticed.^3 ,4^ Consequently, the number of thyroidectomies performed has risen, as surgical intervention is often essential in malignant cases. Despite existing literature advocating less invasive diagnostic and treatment approaches for thyroid diseases, this shift has not been significantly adopted.^5^

 In Brazil, Braga et al.^6^ reported 160,219 thyroidectomies performed in the public health system between 2010 and 2020. Comparatively, 871,644 patients underwent thyroidectomy in the United States between 1993 and 2008.^7^ Thyroidectomy is associated with low in-hospital mortality rates, ranging from 0.03% to 0.61%.^8-10^ Thus, most studies have focused on complications such as recurrent laryngeal nerve injury, hypoparathyroidism, and reoperations, rather than mortality. Although the mortality risk is minimal, given the high frequency of this procedure, analyzing mortality outcomes remains crucial. Notably, comprehensive studies providing normative data on thyroidectomy outcomes at the national level are lacking. 

 The relationship between hospital volume and complication rates in thyroidectomies has received considerable attention. Several studies have demonstrated that higher hospital and surgeon volumes are correlated with better surgical outcomes.^9,11^ However, these findings have not been thoroughly explored in Brazil. In this study, we analyzed the profile of thyroidectomies performed in the Brazilian public health system from 2008 to 2023 to identify factors associated with in-hospital mortality. 

## METHODS

 A retrospective, quantitative, and descriptive approach was applied using aggregated population data. All information related to the surgical procedures was extracted from the public page of the Department of Information Technology of the Unified Health System (DATASUS),^12^ a government digital platform that provides open-access data on procedures performed in the public health sector. Data were collected from 2008 to 2023. 

 The procedures were described in codes, and the following codes related to thyroidectomies were included in the analysis:

- Partial thyroidectomy (04.02.01.003-5)
- Total thyroidectomy (04.02.01.004-3)
- Total thyroidectomy with neck dissection in oncology (04.16.03.012-2)
- Total thyroidectomy with neck dissection (04.02.01.005-1)
- Total thyroidectomy in oncology (04.16.03.013-0 and 04.16.03.027-0)

The analyses included a quantitative evaluation of the total number of procedures, average hospital stay, number of deaths, amount reimbursed to healthcare facilities for the procedure, and the nature of hospitalizations. Demographic data were analyzed to evaluate the geographical distribution of surgeries across Brazilian states, patient flow between federative entities, and concentration of procedures in destination states. The main indications for surgical procedures were identified using the International Classification of Diseases codes associated with each Hospital Admission Authorization. 

 The number of head and neck surgical specialists was obtained from data provided by the Brazilian Medical Association.^13^ Hospitals were stratified into quartiles based on the total number of procedures performed. This approach was adopted to classify facilities as high-, intermediate-, low-, or very low- volume institutions within our dataset, rather than relying on fixed thresholds established in the literature. This stratification allowed for a more context-specific assessment of surgical volume. In this stratification, hospitals in the high-, intermediate-, low-, and very low-volume groups performed > 98, 57–97, 25–56, and < 25 procedures each year, respectively. 

 Statistical analyses were conducted using Jamovi software (The Jamovi Project, Australia, 2022). The Kruskal–Wallis test was used to compare multiple groups, followed by the Dwass–SteelCritchlow–Fligner test for post hoc comparisons. The Spearman’s correlation test was used to evaluate relationships between quantitative variables, and the Mann–Whitney U test was used for two-group comparisons. Non-parametric methods were selected because of their robustness in analyzing aggregated and non-normally distributed data. 

 The Institutional Review Board of the Hospital Israelita Albert Einstein. approved this study (approval number: 6010). All data were anonymized and publicly accessible, ensuring compliance with ethical and privacy standards. Data extraction and cleaning procedures followed the Reporting of studies Conducted using Observational Routinely-collected Data statement. 

## RESULTS

 Between 2008 and 2023, a total of 230,345 thyroidectomy procedures performed across 1,383 healthcare facilities were registered under the selected Brazilian public Unified Health System (SUS) codes. Of these, 205,316 (89.1%) were performed on in female patients. The mean age of patients at the time of surgery was 46.5 ± 9.9 years. The amount paid by the SUS per hospitalization was BRL 1,209.85, ranging from BRL 528.43 in Acre to BRL 1,707.37 in Rio Grande do Norte. 

 With regard to surgical diagnosis, the most common ICD code was C73 (malignant neoplasm of the thyroid), accounting for 84,474 procedures (36.7%), followed by D44 (neoplasm of uncertain behavior of the thyroid) in 54,190 procedures (23.5%) and D34 (benign neoplasm of the thyroid) in 51,332 procedures (22.2%). 

**Figure 1:**
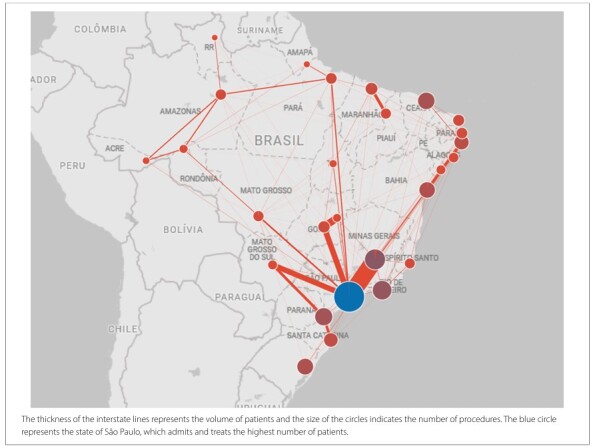
Illustrative map of patient referral flows for thyroidectomy procedures within the SUS across Brazil’s Federal Units from 2008 to 2023.

 When analyzing Brazilian federative units (FUs), Rio Grande do Norte recorded the highest number of procedures (160 procedures/100,000 inhabitants) during the study period, followed by Ceará (152 procedures/100,000 inhabitants). In contrast, the lowest rates were observed in Goiás (66 procedures/100,000 inhabitants) and Pará (51 procedures/100,000 inhabitants). Overall, 49.7% of the procedures were performed outside the patients’ municipality of residence and 14.9% were conducted outside their state of origin. The Brazilian FUs with the highest percentages of procedures performed within their states of origin were São Paulo (99.9%) and Ceará (99.8%). Conversely, FUs with the highest patient referral rates to other states were Goiás (90.8% of procedures performed within the state) and Mato Grosso do Sul (88.8%). Descriptive data of FUs are detailed in **Table 1**, and patient flow between states of origin and procedure-performing states is illustrated in **Figure 1**. 

**Table 1:** General description of thyroidectomy procedures performed in the Brazilian public Unified Health System (SUS) by federal unit from 2008 to 2023

**State of origin**	**Procedures performed(n)**	**Establishments (n)**	**Procedures/establishment (n)**	**Establishments/ 100.000 people (n)**	**% Procedures Performed Within the State**	**Procedure Specialists per 100,000 people**	**Number of Specialists**	**Specialists per 100,000 people**	**Mortality**
Acre	1,200	4	300	0,45	95.96	136.27	4	0.45	0.08
Alagoas	3,509	19	184.68	0,59	98.35	108.97	17	0.53	0.14
Amapá	859	4	214.75	0,5	98.2	107	2	0.25	0.35
Amazonas	4,173	16	260.81	0,37	98	97.47	16	0.37	0.24
Bahia	12,394	107	115.83	0,72	98.25	83.46	72	0.48	0.08
Ceará	14,032	55	255.13	0,6	99.86	151.97	60	0.65	0,09
Distrito Federal	2,522	15	168.13	0,5	98.75	84.55	39	1.31	0.12
Espírito Santo	3,887	32	121.47	0,78	99.25	94.76	30	0.73	0
Goiás	4,887	57	85.74	0,78	90.86	66.49	32	0.44	0.18
Maranhão	5,385	51	105.59	0,73	97.63	76.81	21	0.3	0.11
Mato Grosso	4,006	29	138.14	0,76	98.21	104.42	20	0.52	0.1
Mato Grosso do Sul	2,670	14	190.71	0,48	88.8	92.01	17	0.59	0.11
Minas Gerais	21,941	179	122.58	0,84	96.8	102.9	97	0.45	0.2
Pará	4,427	35	126.49	0,4	97.01	51.09	11	0.13	0.34
Paraíba	5,178	30	172.6	0,72	99.24	124.92	29	0.7	0.08
Paraná	15,549	91	170.87	0,77	99.67	131.5	69	0.58	0.18
Pernambuco	10,642	31	343.29	0,32	98.98	111.56	51	0.53	0.06
Piauí	4,473	22	203.32	0,65	99.58	132.51	14	0.41	0.16
Rio de Janeiro	18,715	128	146.21	0,74	99.8	108.68	76	0.44	0.17
Rio Grande do Norte	5,517	16	344.81	0,46	99.65	160.1	24	0.7	0.07
Rio Grande do Sul	12,245	107	114.44	0,95	99.79	109.04	52	0.46	0.23
Rondônia	2,149	8	268.63	0,46	98.26	123.07	6	0.34	0.19
Roraima	622	2	311	0,28	95.74	86.78	3	0.42	0.32
Santa Catarina	8,849	78	113.45	0,97	98.68	109.81	51	0.63	0.09
São Paulo	55,956	238	235.11	0,52	99.91	121.71	568	1.24	0.16
Sergipe	3,035	6	505.83	0,26	99.61	132.47	17	0.74	0.13
Tocantins	1,523	9	169.22	0,57	97.64	96.55	8	0.51	0.59

 In total, 352 in-hospital deaths were recorded during the study period, corresponding to an overall in-hospital mortality rate of 0.15%. FUs with the highest in-hospital mortality rates were Tocantins (0.6%) and Amapá (0.35%), whereas the lowest rates were observed in Espírito Santo, which reported no in-hospital deaths, and in Pernambuco (0.06%). 

 Head and neck surgery is the primary specialty performing thyroidectomies in Brazil, with 0.69 specialists per 100,000 inhabitants. Geographically, São Paulo (1.2 specialists per 100,000 inhabitants) and the Federal District (1.3 specialists per 100,000 inhabitants) had exhibit the highest proportions, whereas Amapá (0.25 specialists per 100,000 inhabitants) and Pará (0.13 specialists per 100,000 inhabitants) had have the lowest number. This represents a nearly fivefold difference between the highest- and lowest-density regions (1.3 versus 0.13 specialists per 100,000 inhabitants) and a fourfold difference between São Paulo and Amapá (1.2 versus 0.25). These variations reflect substantial disparities in specialist availability across countries. 

**Figure 2:**
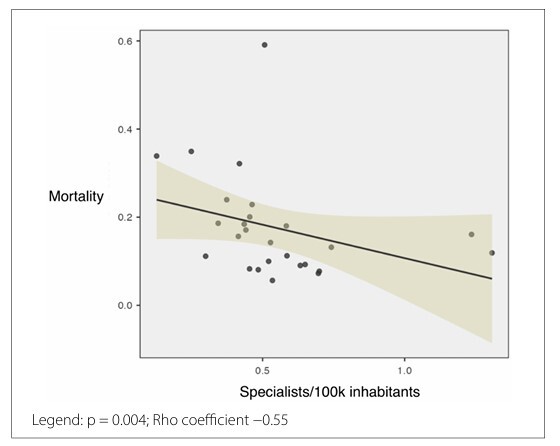
Scatter plot illustrating the correlation between in-hospital mortality for thyroidectomy admissions and the rate of head and neck surgeons per 100.000 inhabitants in Brazil.

 Spearman’s correlation test was conducted to identify the variables associated with higher mortality rates. We observed a significant negative correlation between the mortality rate and both the number of procedures per 100,000 inhabitants (p = 0.046; Rho coefficient −0.4) and number of specialists per 100,000 inhabitants (p = 0.004; Rho coefficient −0.55). However, no significant correlation was noted for the number of procedures per facility, facilities per 100,000 inhabitants, or the percentage of procedures performed within the state (p = 0.5; p = 0.8; p = 0.2, respectively). The correlation between mortality and the number of head and neck surgeons per 100,000 inhabitants is presented in **Figure 2**. 

 An exploratory analysis was conducted by testing thresholds in increments of 0.1 specialists per 100,000 inhabitants to determine the cutoff value at which the differences in mortality ceased to be significant. A threshold of 0.3 specialists per 100,000 inhabitants was not associated with significant differences in mortality (Mann–Whitney U test, p = 0.18). However, a cutoff of 0.5 specialists per 100,000 inhabitants marked the point at which a significant difference in mortality was observed (Mann–Whitney U test, p = 0.02). At this threshold, the mean mortality rate was 0.20 in the group with up to 0.5 specialists per 100,000 inhabitants compared to 0.15 in the group with more than 0.5 specialists per 100,000 inhabitants, representing a 25% reduction. 

 A total of 1,383 healthcare facilities performed thyroidectomies during the study period. Of these, 1,109 facilities (80.2%) performed fewer than 12 procedures annually, accounting for 32,070 procedures (13.9% of the national total). Facilities performing only 1 procedure annually accounted for 1.3% of the procedures; 25.7% (59,587 procedures) were conducted in facilities performing up to 25 procedures per year, and 34.8% (80,183 procedures) in facilities performing more than 76 procedures per year. 

**Table 2** provides comparative data for groups of facilities stratified by procedure volume regarding in-hospital mortality, the average amount paid for SUS hospitalization, length of stay, and percentage of hospitalizations involving intensive care unit (ICU) use. Significant differences were observed among the groups for all analyzed variables (Kruskal–Wallis test). Multiple comparisons using the Dwass–Steel–Critchlow–Fligner test revealed significant differences between the very low-volume and high-, intermediate-, and low-volume groups. The very low-volume group had lower hospitalization costs, higher percentage of ICU hospitalizations, and higher mortality rates. The mortality rates were 0.51%, 0.13 %, 0.14 %, and 0.1% in the very low-, low-, intermediate-, and high-volume groups, respectively. This difference was significant only when comparing the very low-volume group with the other groups, whereas no significant differences were observed among the low-, intermediate-, and high-volume groups. 

**Table 2:** Comparison of in-hospital mortality, amount paid by the Brazilian public Unified Health System (SUS), length of stay, and ICU utilization across facility volume groups

**Group**	**High Volume (HV)**	**Intermediate volume (IV)**	**Low volume (LV)**	**Very low volume (VLV)**	**p (Kruskal-Wallis)**	**DSCF Multiple Comparisons**
Hospitalizations/year, (n)	150.8 ± 52,3	72.8 ± 11.7	35 ± 8,3	3 ± 5.1		
Average amount paid by SUS per hospitalization (n)	1564.08 ± 694.8	1143.5 ± 577.7	1107,6 ± 555,9	744.5 ± 745	< 0.001	*HV × IV p = 0.082*
						*HV × LV p = 0.22*
						*HV × VLV p = <0.001*
						*IV × LV p = 0.98*
						*IV × VLV p = <0.001*
						*LV × VLV p = <0.001*
Mortality rate (%)	0.1 ± 0.12	0.14 ± 0.16	0,13 ± 0,16	0.51 ± 4.36	< 0.001	*HV × IV p = 0.954*
						*HV × LV p = 1*
						*HV × VLV p = <0.001*
						*IV × LV p = 0.95*
						*IV × VLV p = <0.001*
						*LV × VLV p = <0.001*
Average length of stay (n)	2.3 ± 0.76	2.77 ± 1.49	2,58 ± 1,47	2.60 ± 2.63	0.018	*HV × IV p = 0.526*
						*HV × LV p = 1*
						*HV × VLV p = 0.91*
						*IV × LV p = 0.304*
						*IV × VLV p = 0.022*
						*LV × VLV p = 0.53*
Percentage of Hospitalizations with use of ICU (n)	2.08 ± 1.15	2.29 ± 2	2,74 ± 2,38	4.7 ± 15	< 0.001	*HV × IV p = 0.997*
						*HV × LV p = 0.907*
						*HV × VLV p = <0.001*
						*IV × LV p = 0.732*
						*IV × VLV p = <0.001*
						*LV × VLV p = <0.001*

ICU, intensive care unit; DSCF, Dwass–Steel–Critchlow–Fligner.

 After determining that higher mortality rates were associated with facilities performing very few procedures, we conducted a subgroup analysis that excluded facilities performing fewer than 10 procedures annually. The remaining facilities were re-stratified into quartiles based on the procedure volume. This exclusion accounted for 12% of procedures and 78.4% of facilities. Comparative analysis of the quartiles in this subgroup showed no significant differences in mortality rates (Kruskal–Wallis test, p = 0.3). 

## DISCUSSION

 The Brazilian healthcare system operates within a dual structure, encompassing both the public and private sectors, each with distinct roles and contributions to the overall healthcare landscape. Only 28.5% of the population has access to private healthcare, whereas the remaining individuals rely exclusively on the public healthcare system.^14,15^ In this study, we analyzed only thyroidectomies performed within the public healthcare system. 

 Although the volume of thyroid surgeries is widely recognized as an important factor in the literature, it remains poorly studied in Brazil. This has led to discussions based on the limited availability of supporting data.^16,17^ The present study provides valuable new evidence regarding thyroidectomies performed in Brazil, specifically addressing hospital surgical volumes, distribution of specialists, and in-hospital mortality rates. 

 Recently, the number of thyroidectomies performed by the Brazilian public health system has remained relatively stable. Braga et al.^6^ described the profile of thyroidectomies performed by the SUS between 2010 and 2020. Their findings indicated a significant decrease in procedures during the coronavirus disease 2019 pandemic in 2020; however, the volume stabilized at approximately 15,000 procedures annually for the remainder of the study period. They also discussed mortality rates and regional disparities within Brazil, noting that the highest mortality rate was reported in the northern region. However, they did not analyze other variables that may have influenced mortality, which were addressed in the present study. The overall mortality rate of 0.15% reported in our series aligns with those of Braga et al.^6^ and with international literature, which reported in-hospital mortality rates ranging from 0.03% to 0.61%.^8,9^

 In Brazil, the medical specialty responsible for performing thyroidectomies is head and neck surgery; however, this varies across countries. This specialty is among the 10 least common among the 55 recognized specialties in the country, with only 1,406 specialists registered in 2022. This number has significantly increased over the past 10 years, more than doubling from 631 specialists since 2012.^13^ However, this growth has been insufficient and is further compounded by poor distribution across Brazil. For example, São Paulo had 568 specialists, whereas the entire state of Amapá had only 2. Access to specialists is often further hindered by the vast size of some states and the highly dispersed population in large areas, such as Amazonas and Pará. Specialists are primarily concentrated in the state capitals, exacerbating the challenges of accessing specialized care. Therefore, thyroidectomies are frequently performed by nonspecialists. 

 This study identified a negative correlation between the number of head and neck surgery specialists in Brazil and perioperative mortality rates. To the best of our knowledge, this association has not been established. The cut-off for this analysis was set at 0.5 specialists per 100,000 inhabitants, and approximately half of Brazil’s states^13^ did not meet this threshold. 

 However, this relationship reflects an ecological rather than causal association. Because DATASUS lacks surgeon-level identifiers and specialty information, the link between lower specialist density and higher mortality should be interpreted as an inference drawn from aggregated data and not as a directly measured finding. Moreover, as with any ecological association, the correlations observed at the state level may not accurately represent the individual-level risk. Nevertheless, the unequal geographical distribution of the specialists provides a coherent contextual rationale for the identified mortality patterns. Similarly, Stopenski et al.^18^ analyzed the discrepancies in thyroidectomy outcomes between general surgeons and otolaryngologists specializing in thyroid surgery. Their findings indicated that specialized training led to better surgical outcomes, reinforcing the importance of the specialists involved in these procedures. 

 Data on thyroidectomy procedures analyzed in this study were extracted from DATASUS, the official and mandatory nationwide platform for hospital reimbursements in the SUS. Although regional inequalities in healthcare infrastructure are well recognized, the coding and reporting processes are standardized, minimizing systematic discrepancies across states. Nevertheless, the absence of surgeon-level information is a critical limitation. In regions with a very low specialist density, some thyroidectomies might have been performed by non-specialists; however, this factor could not be directly assessed in our dataset. Therefore, while specialist availability may contribute to regional disparities, the most consistent determinant of higher mortality was hospital surgical volume, particularly in very low-volume centers. 

 Moreover, recent studies have emphasized a global trend toward deintensified screening and treatment of thyroid diseases,^19^ which may further reduce thyroidectomy rates over time. If this decline extends to Brazil, both individual and institutional surgical experience may decrease, potentially amplifying the risks associated with low-volume practices. Therefore, continuous surgical training and careful centralization of thyroid surgery are essential to maintain safety standards. 

 Surgical volume is a well-established factor associated with surgical quality and safety, both in general^20,21^ and specifically in thyroid surgery.^11^ Theodor Kocher was the first to demonstrate a significant reduction in operative mortality with increased surgical experience.^22^ Although reports vary on the specific number of surgeries defining high and low volume, the consensus is that high-volume surgeons tend to have fewer complications.^7,11^ Additionally, thyroidectomies performed by high-volume hospitals and surgeons also result in shorter hospital stays and lower costs compared to those performed by low-volume hospitals and surgeons.^9,23^ However, most studies have focused on long-term complications rather than in-hospital mortality, given the latter’s low frequency, and studies that have described mortality rates do not always show differences according to volume.^24^

 In complex procedures such as pancreatectomy and liver transplantation, the literature strongly supports public policies that discourage surgeries at very low-volume hospitals. High-volume centers are consistently associated with better patient outcomes, including lower complication and mortality rates.^25^ Policymakers are encouraged to incorporate these findings into strategies to optimize surgical care and ensure patient safety.^26,27^ However, this relationship is less well established for mortality rates following thyroidectomies, particularly in Brazil, where limited data exist on the subject. 

 In this study, we focused on in-hospital mortality and found that very low-volume hospitals (those performing fewer than 25 thyroidectomies annually) were associated with significantly higher mortality rates than all other groups. This finding underscores the role of very low-volume hospitals as a key factor associated with increased mortality, with mortality rates five times higher than those of high-volume hospitals. Notably, when hospitals performing fewer than 10 thyroidectomies annually were excluded from the analysis, the mortality differences among the volume categories declined, representing 12% of procedures and 78.4% of establishments. These findings are highly relevant from a public health perspective, demonstrating that although thyroidectomy is not a highly complex procedure with elevated mortality, health systems could benefit from policies that restrict such procedures in very low-volume centers. Establishing a threshold of at least 10 thyroidectomies per year seems to offer meaningful benefits, particularly for outcomes as critical as in-hospital mortality. 

 Although the data analyzed in this study did not allow the evaluation of individual surgeon volume or nonfatal complications such as laryngeal nerve injury or hypoparathyroidism, we can reasonably infer that hospital volume largely reflects surgeon volume for thyroidectomies. Some authors argue that the favorable impact of hospital volume on surgical outcomes is explained by surgeon volume, especially for procedures requiring shorter stays and less dependence on hospital-based resources.^22^ Thyroid surgery exemplifies such a procedure, where surgeon experience significantly impacts outcomes, and has been recognized for nearly a century. 

 This study has some limitations, mainly inherent to the use of secondary data. Information on the identity and specialty of the surgeon and postoperative complications beyond in-hospital mortality was not available, and data from the private healthcare system were not included. Moreover, the observed correlations should be interpreted as ecological associations rather than causal relationships, given the absence of individual-level data. Despite these limitations, this analysis provides nationwide evidence on the relationship between hospital volume and perioperative mortality in thyroidectomies, an aspect rarely explored in Brazil. These findings reflect the information currently available in national health databases and offer a valid contribution to understanding regional disparities and guiding future policy development. 

 Our results can help guide practical action to improve the care and quality of service provided in public health services. Creating regional flows in each state to direct potential surgical cases to hospital services with a capacity and volume exceeding 10 procedures per year could improve care. Creating a proposal with regulatory bodies for surgical procedures in Brazil, such as the High-Complexity Care Regulation System, with well-established flows, could be a viable alternative to optimize resources and offer high-quality health services for diseases within the specialty of Head and Neck Surgery. 

## CONCLUSION

 Thyroidectomy has a low overall perioperative mortality rate; however, our data showed a significant increase in mortality in very low-volume hospitals. This difference disappeared when hospitals with fewer than 10 surgeries were excluded from the analysis. 

 Therefore, the centralization of thyroid surgery in hospitals that perform at least 10 cases annually may represent a cost-effective strategy to reduce preventable deaths and optimize resource allocation within Brazil’s public health system. 

## Data Availability

The datasets analyzed during the current study are available in the DATASUS repository ( http://tabnet.datasus.gov.br).
